# Five steps to high quality antimicrobial stewardship research

**DOI:** 10.1017/ash.2024.73

**Published:** 2024-05-09

**Authors:** Bradley J. Langford, Pamela Bailey, Daniel J. Livorsi, Kevin A. Brown, Sonali D. Advani, Elizabeth Dodds Ashley, Gonzalo Bearman, Priya Nori

**Affiliations:** 1 Public Health Ontario, Toronto, Ontario, Canada; 2 Dalla Lana School of Public Health, University of Toronto, Toronto, Ontario, Canada; 3 Prisma Health, Columbia, SC, USA; 4 Center for Access and Delivery Research and Evaluation (CADRE), Iowa City Veterans Affairs Health Care System, Iowa City, IA, USA; 5 Division of Infectious Diseases, University of Iowa Carver College of Medicine, Iowa City, IA, USA; 6 Duke University School of Medicine, Durham, NC, USA; 7 Division of Infectious Diseases, Duke University School of Medicine and Duke Center for Antimicrobial Stewardship and Infection Prevention, Durham, NC, USA; 8 Healthcare Infection Prevention Program, Virginia Commonwealth University Health System, Richmond, VA, USA; 9 Department of Medicine, Division of Infectious Diseases, Montefiore Medical Center, Bronx, NY, USA

## Abstract

The escalating threat of antimicrobial resistance (AMR) necessitates impactful, reproducible, and scalable antimicrobial stewardship strategies. This review addresses the critical need to enhance the quality of antimicrobial stewardship intervention research. We propose five considerations for authors planning and evaluating antimicrobial stewardship initiatives. Antimicrobial stewards should consider the following mnemonic ABCDE: (A) plan **A**head using implementation science; (B) **B**e clear and thoroughly describe the intervention by using the TidIER checklist; (C) Use a **C**hecklist to comprehensively report study components; (D) Select a study **D**esign carefully; and (E) Assess **E**ffectiveness and implementation by selecting meaningful outcomes. Incorporating these recommendations will help strengthen the evidence base of antimicrobial stewardship literature and support optimal implementation of strategies to mitigate AMR.

## Introduction

Antimicrobial overuse is fueling the growing health threat of antimicrobial resistance (AMR). Strategies to use antimicrobials more judiciously—increasing the appropriateness of initiation, selection, and duration—are crucial to conserve the effectiveness of these life-saving medications.^1^ While antimicrobial stewardship is a core component of global and national action plans to mitigate AMR, there is a need to identify optimal interventions and implementation approaches.

Over the past three decades, the number of research articles on the topic of antimicrobial stewardship has increased exponentially, with over 10,000 indexed articles on Medline to date.^2^ However, the quality of most antimicrobial stewardship intervention studies is notably low and has not improved over this period.^3^ Many studies are single center, lack control groups, and are observational in nature. While national guidelines aim to inform the implementation of antimicrobial stewardship interventions across healthcare settings, the vast majority of guideline recommendations are based on low-quality evidence and are weak recommendations.^4^ A Cochrane systematic review of interventions to improve antimicrobial prescribing in hospitalized patients found that there was high certainty evidence that such interventions improve the appropriateness and reduce the volume of antimicrobial prescribing.^5^ However, few studies evaluated clinical outcomes like AMR, for which mitigation is the main impetus for this work. The risk of bias for most antimicrobial stewardship intervention studies was high and the quality of reporting details of interventions was poor, which can compromise reproducibility and efforts to scale them up across wider regions.^5^ Further, most of these antimicrobial stewardship interventions were not informed by behavioral science, suggesting the effectiveness of such approaches can be further improved.

Improvements in the quality of antimicrobial stewardship intervention and implementation research can support greater recognition of the importance of antimicrobial stewardship in mitigating AMR, as well as improve reproducibility and scalability across settings. Achieving a higher quality will also increase the likelihood of publication in reputable journals, an important aspect of knowledge dissemination and translation.^6^ We aim to provide five considerations to improve the quality and impact of antimicrobial stewardship research (see Table 1).

**Table 1.** ABCDE: A Simple Blueprint for Antimicrobial Stewardship Intervention Research

□	**A: Plan Ahead Using Implementation Science** ^7^ (Use at least one implementation framework to inform the intervention) □ Process framework (eg, Iowa Model, QUERI) □ Determinant framework (eg, CFIR, iPARIHS, and TDF) □ Other, specify: _________ □ Not applicable, specify reason:_____________________________
□	**B: Be Clear and “Tidy” about Your Intervention** ^8^ □ Follow the Template for Intervention Description and Replication (TIDieR)
□	**C: Use a Checklist to Comprehensively Report Study Components** ^9^ (select one) □ RCT: CONSORT statement □ Observational: STROBE statement □ Outbreak or intervention study for nosocomial infection: ORION □ Qualitative: COREQ □ Other, specify: _________ □ Not applicable, specify reason:___________________________
□	**D: Select Study Design Carefully** ^10^ (select one) □ Individual RCT OR □ cluster RCT □ Quasi-experimental □ Account for time trends (use interrupted time series with segmented regression) □ Include control group (eg, control drug, ward, or hospital) □ Measure and report any changes in patient population before and after □ Other, specify: _________
□	**E: Measure Effectiveness by Selecting Meaningful Outcomes** **(select as many as possible)** □ Consider using the RE-AIM framework to guide your evaluation □ Measure relevant implementation outcomes □ Report confidence intervals in lieu of/in addition to p-values □ Antimicrobial use measures □ Include specific classes and total antimicrobial use where possible □ Clinical outcomes; specify: ____________________ □ Antimicrobial resistance; specify: ____________________

RE-AIM stands for Reach, Effectiveness, Adoption, Implementation, and Maintenance.

## Plan Ahead using implementation science

Many antimicrobial stewardship interventions were conceived based on the ISLAGIATT principle, a tongue-in-cheek acronym for “It seemed like a good idea at the time” coined by Martin Eccles at the University of Newcastle. ASP interventions are often informed by researchers’ previous experience, a gestalt of what they expect will work in their setting, and existing published work or experience from other settings. However, the field of implementation science offers several tools to ensure interventions are selected in a thoughtful manner and are more likely to account for the barriers and enablers specific to a local context.^7^ Use of an implementation science framework is highly recommended during this planning phase, and we mention several potentially pertinent frameworks in the paragraphs below.

### Identify the evidence-practice gap

An initial investment in planning the ASP intervention can yield benefits by increasing the likelihood of success. Several implementation process frameworks, such as the Iowa Model or the Quality Enhancement Research Initiative roadmap, provide a step-by-step guide for planning and carrying out a new intervention.^11,12^ In many of these frameworks, the first step in designing an appropriate intervention is to clearly articulate the evidence-practice gap that exists and to specify the desired behavior change. Researchers should clearly indicate *who* needs to do *what* differently, *where*, *when* and *by how much* (ie, state the AACTT: action, actor, context, target, and time).^13^ This provides a foundation to inform subsequent steps in the process.

### Assess organizational climate and engage end-users

Across each stage, end-users should be engaged in the process to ensure their needs, preferences and priorities are taken into account in the intervention’s design. The concept of integrated knowledge translation (iKT) suggests that end-users should be integrated into the research process as early as possible and kept engaged along the way.^14^ The “IKEA effect” posits that people value things more if they made them themselves.^15^ As such, participants involved in the development of ASP initiatives later become champions during the roll-out phase. Assembling a diverse research team including patient partners can help reduce risks of bias (towards age, sex/gender, ethnicity, etc) and support a more equitable study design to ensure all those who are eligible have an opportunity to participate.^16^

### Identify barriers and facilitators to success

Not all ASP strategies work in all settings. For example: shared decision-making may be more appropriate in primary care but out of place in a hospital ICU; prospective audit and feedback may be well-suited for acute care hospitals but a poor fit in community settings. Understanding the unique contextual factors can influence the selection of optimal antimicrobial stewardship strategies. Sources of data to understand these contextual determinants, barriers and facilitators to behavior change include existing literature, surveys, focus groups, and one-on-one interviews. Selecting a determinant framework can help to conceptually organize and better understand barriers and facilitators. A commonly used and widely applicable framework is the Theoretical Domains Framework (TDF), a list of 14 constructs that can be used to classify barriers and facilitators, such as social influences, knowledge, emotions, and beliefs about consequences.^17^ The Consolidated Framework for Implementation Research (CFIR) is another determinant framework that includes five domains that influence implementation: the outer (eg, values, policies) and inner (eg, culture, resources) settings, individuals (eg, needs, motivations), the innovation (the “thing” being implemented), and the implementation process (eg, engaging, adapting).^18^

### Choose an implementation strategy

While the desired *evidence-based practic*e is “the thing” antimicrobial stewards aim to implement, the *implementation strategy* is the approach used “to try to help people and places ‘do the thing’.”^7^ Most implementation strategies are multi-modal and should be selected based on the known barriers and facilitators to achieving a desired practice change, as identified through the planning process. Typically, a bundle of implementation strategies are chosen. Various online mapping tools exist to help researchers select appropriate strategies based on existing frameworks, such as the StrategEase Tool^19^ using the TDF and the Implementation Strategy Selection Tool for CFIR.^18^ This approach to theory-informed selection of interventions may help to identify feasible and impactful strategies for a given context.

## Be clear and “tidy” about your intervention

A complete description of interventions being evaluated is an essential component of research; however, it is often limited in publications which therefore limit reproducibility and does not allow others to build on the existing work. A cross-sectional study of papers published in *BMJ* noted that 57% trials included insufficient descriptions to allow replication.^20^ In an attempt to rectify this, the Template for Intervention Description and Replication (TIDieR) was developed.^8^ This checklist contains the minimum recommended items for describing an intervention and is broadly applicable to most ASP interventions.

The TIDieR checklist is viewed as an extension of the PICO (Patient, Intervention, Comparator, Outcome) as a guide to reporting the main elements of the research question. The checklist is available in template format and in multiple languages and should be included in supplemental material or appendices of the research manuscript.

The authors of TIDieR recommend inclusion into peer review and publication standards. Since its introduction in 2014, TIDieR has been adapted into population health,^21^ placebo and sham controls,^22^ telehealth^23^ and systematic review^24^ iterations or adaptations.

## Use a Checklist to comprehensively report study components

Robust antimicrobial stewardship studies include transparent and comprehensive reporting of their rationale, objective, methodology, results, limitations, and implications. Transparent reporting helps to facilitate: (1) the reader’s understanding of the study; (2) use of findings to inform decision-making; (3) replication by other researchers; and (4) incorporation and synthesis in systematic reviews. To help facilitate reporting, several guidelines with easy-to-use checklists tailored to specific study designs are available from the Equator Network (Enhancing the QUAlity and Transparency Of health Research).^9^ Commonly used checklists for antimicrobial stewardship studies may include, but are not limited to, the CONSORT statement (Consolidated Standards of Reporting Trials for randomized controlled trials,^25^ ORION statement (guidelines for reporting of outbreak reports and interventions studies of nosocomial infection),^26^ and STROBE-AMS (Strengthening the Reporting of Observational Studies in Epidemiology) for observational epidemiological studies evaluating the impact of antimicrobial use on resistance.^27^

## Select study Design carefully

The Cochrane review on antimicrobial stewardship interventions in hospitals suggests that further studies comparing antimicrobial stewardship to no intervention will add minimal value to the body of literature on this topic, since it is clear there is a benefit to these programs.^5^ Instead, studies should aim to compare different antimicrobial stewardship approaches to each other and improve their sustainability and scalability.

Because stewardship interventions often target providers, inpatient units, clinics or entire healthcare institutions, and outcomes are measured at the patient level, many stewardship studies involve clustered outcomes. Parallel arm cluster RCTs are the ideal and highest quality design for antimicrobial stewardship interventions since they provide benefits due to randomization and control for secular and seasonal trends. Seasonality, in particular is very frequently noted in antimicrobial prescribing, driven by viral respiratory pathogens that circulate at increased rates in the winter months.^28^ Conduct of a RCTs should not be dismissed out of hand, since the quality of evidence is disproportionately stronger, and these studies, in some instances, can be quite affordable to conduct.

Due to ease of implementation and their observational nature, uncontrolled before-and-after studies are often employed to estimate the impact of antimicrobial stewardship interventions. However this approach is prone to bias because uncontrolled before-and-after studies do not account for secular and seasonal time trends occurring irrespective of the intervention (for example a decline in antimicrobial use already occurring may be incorrectly attributed to an intervention). The Joint Programming Initiative on Antimicrobial Resistance (JPIAMR) Working Group on Design of Antimicrobial Stewardship Evaluations offers recommendations that are relevant to these quasi-experimental studies, and recommends using an interrupted time series (ITS) approach to help estimate the impacts of an intervention over the projected secular time trends, also known as the counterfactual scenario.^10^ Some manuscripts incorrectly refer to before-and-after studies that simply compare the mean level in outcome before and after the intervention as ITS. It is important to note that ITS usually estimates the change in the outcome level and slope after the intervention, compared to the counterfactual.^29^ However there are some key limitations to this approach. ITS is susceptible to the quality of the projected time trends, and because of this, projections far beyond the time of the intervention are problematic. Additionally ITS is susceptible to time-varying confounders, such as other interventions or changes that occurred during intervention period.

A middle ground between a parallel arm cluster RCT, and an uncontrolled ITS, is the controlled ITS design (also known as the difference-in-difference design), which can often enable a more robust evaluation of the longer-term impacts of an intervention compared to ITS design. In this design, a control group is included alongside the intervention group (separate prescribers, hospitals, wards that do not receive the intervention) and allows for a more reliable estimation of patterns that would have occurred in the absence of intervention.^30^ The control group can consist of other prescribers or hospitals that do not receive the intervention, or of an alternate control outcome, such as a drug not expected to be impacted by the outcome. Trends in the two groups prior to the intervention should be similar (though baseline levels could be different), and the estimated effect of the intervention is the relative change in the level of the outcome after the intervention.

## Assess Effectiveness and implementation by selecting meaningful outcomes

### Implementation outcomes

While the focus of most antimicrobial stewardship efforts is to improve the appropriateness of antimicrobial prescribing, improve clinical outcomes, and mitigate AMR, it is recommended to also include process measures that will affect the intervention’s impact, generalizability, and sustainability. One useful framework to plan and evaluate interventions comprehensively is RE-AIM (Reach, Effectiveness, Adoption, Implementation, and Maintenance).^31^ The framework prompts researchers to think broadly, both quantitatively and qualitatively, about several outcomes beyond effectiveness, including the reach (eg, proportion of target population that participated), adoption (eg, the number of eligible settings where the intervention was applied and who applied it), implementation (eg, fidelity, acceptability, costs), and maintenance (eg, how long are the results of the intervention sustained). The RE-AIM framework has been used in at least one prior antimicrobial stewardship intervention.^32^ Further application of this framework will help antimicrobial stewardship researchers balance internal and external validity, thereby producing interventions that are more likely to be adopted and sustained in other settings.

If the RE-AIM framework is not used, many of its outcomes should still be incorporated into the evaluation process, particularly implementation outcomes like acceptability, adaptation, cost, feasibility, fidelity and sustainability. A more detailed discussion of implementation outcomes can be found elsewhere.^7^

### Antimicrobial use outcomes

Antimicrobial use (AU) is often the primary effectiveness outcome for antimicrobial stewardship interventions. However, there are many ways to measure use, which are dependent on the availability of data and the objective of the intervention (whether aim is to improve antimicrobial initiation, selection, or duration). For example, days of therapy (DOT) may be preferred over defined daily doses (DDD), but often requires more sophisticated technology to collect.

To facilitate standardized antimicrobial stewardship outcome measurement in hospitals, Duke Antimicrobial Stewardship Outreach Network (DASON) developed a technical manual suggesting metrics that are both useful and feasible, including days of therapy over patient days, redundant therapy events, total duration per antimicrobial admission, and de-escalation.^33^ Notably in the United States, the Centers for Disease Control and Prevention National Healthcare Safety Network (NHSN) AU module allows hospitals to benchmark their AU data in DOT per 1000 patient days present. Such modules are a valuable source of data for evaluating the impact of ASP interventions.^34^ In outpatient settings, measurement is highly dependent on the healthcare system and information technology infrastructure. Common metrics for community AU may include defined daily doses or days of therapy per inhabitant days, or simply the number of prescriptions denominated by the number of inhabitants or outpatient visits.^35^ While many antimicrobial stewardship interventions target a specific class of antimicrobials (eg reducing unnecessary fluoroquinolone prescribing), care should be taken to also evaluate overall antimicrobial prescribing. Such interventions may result in shifting prescribing from one targeted class to another without improving overall prescribing, a concept coined “squeezing the balloon.”^36^ Additionally, antimicrobial stewardship interventions are ideally set up for negative tracer outcomes. As interventions often target specific medications, non-targeted medications can be used as negative control outcomes and compared to medications targeted by the initiative.

Despite the importance of antimicrobial utilization, the aim of most antimicrobial stewardship initiatives is not to simply reduce utilization, but rather to increase appropriateness of prescribing. However, measurement of appropriateness is challenged by subjectivity in adjudication and labor intensity, which often require time-intensive chart reviews.^33^ Nevertheless, appropriateness should be considered where feasible to evaluate the adequacy of antimicrobial initiation, selection, and duration. The use of pre-existing data in the medical record or administrative data may help to improve the scalability of assessment of appropriateness, particularly for large scale research. For example, outpatient antibiotic prescribing for upper respiratory tract infection can be assessed against diagnostic billing codes to estimate appropriateness.^37,38^ Antimicrobial spectrum indices or scores may also be considered if the aim of the intervention is to improve antimicrobial selection and minimize the use of “broader–spectrum” therapy.^39,40^

### Clinical outcomes

Consideration of outcomes of importance to patients is key. Proactively including patient partners in research design can help identify relevant and meaningful measures.^41^ Such outcomes may include length of stay, hospital readmission, requirement for repeat antimicrobial therapy, adverse events, infection with *C. difficile* or an antimicrobial resistant organism, or mortality. Since there are a variety of possible measures, many of which are competing, use of a composite outcome or ordinal outcomes based on desirability may be considered when evaluating patient-level outcomes. Desirability Of Outcome Ranking (DOOR) and Response Adjusted for Duration of Antibiotic Risk (RADAR) are ordinal measures ranking various outcomes from most desirable to least desirable (eg, clinical benefit, clinical benefit with side effects, no benefit, no benefit with side effects, death).^42^ Such approaches can allow for ease of interpretation and a shift of trial design from non-inferiority to superiority which allows for a smaller sample size while maintaining statistical power.

The ultimate objective of antimicrobial stewardship initiatives is to improve patient and population health outcomes, particularly to mitigate antimicrobial resistance. However, few studies have evaluated the impact of such interventions on antimicrobial resistance, and the evidence that does exist is largely lacking in quality.^5,43^ Although individual studies are generally underpowered to detect changes in antimicrobial resistance, given the fundamental importance of this outcome, an assessment for colonization or infection with antimicrobial resistant infections should be included, where possible, in antimicrobial stewardship research.^10^ A report on the global burden of antimicrobial resistance^44^ and the WHO Priority Pathogens list^45^ illustrate potential antimicrobial resistant organism phenotypes for tracking. Large scale ecological studies of antimicrobial stewardship interventions may be best suited to evaluate such outcomes. There is heterogeneity in reporting antimicrobial resistance outcome measures which hinders data synthesis and comparisons.^46^ Incidence rates (eg, counts of extended-spectrum beta-lactamase producing Enterobacterales per 1000 patient days) are commonly reported and may allow for more generalizability than simply reporting proportions (eg, proportion of Enterobacterales that are ESBL producers).

## Statistical analyses

When reporting quantitative outcomes, whether they be antimicrobial use, resistance or process measures, it is important to estimate the degree of uncertainty in the findings, particularly any changes in the outcomes that may have occurred after the intervention. Many statistical experts favor the use of confidence intervals in lieu of p-values. Confidence intervals allow the reader to better understand the precision of the estimate and the full range of possible effect sizes.^47,48^ It is also important to account for multiple hypothesis testing, where possible, by using appropriate statistical tests,^49^ and avoid selectively reporting only results that have achieved statistical significance. Since patient populations may change over time, it may be valuable to report and account for changes in case-mix during the study period. At minimum, studies should account for changes in the population, if any, and include patient days or other population metrics as part of the denominator to account for fluctuations in patient volume.

## Conclusion

Mitigating the threat of antimicrobial resistance requires high quality, impactful, and reproducible antimicrobial stewardship intervention research. To achieve this objective, authors should consider the use of implementation science theories, models, and frameworks, to plan and evaluate their intervention, use of checklists to ensure clear and transparent reporting, selecting a robust study design, and including meaningful process and clinical outcomes to evaluate the impact of their work.
